# Sarcopenia as a Preoperative Risk Stratification Tool among Older Adults with Inflammatory Bowel Disease

**DOI:** 10.20900/agmr20240003

**Published:** 2024-05-30

**Authors:** Ria Minawala, Adam S. Faye

**Affiliations:** 1 Department of Medicine, NYU School of Medicine, New York, NY 10016, USA; 2 Inflammatory Bowel Disease Center, Division of Gastroenterology, Department of Medicine, NYU School of Medicine, New York, NY 10016, USA

**Keywords:** IBD, muscle, surgery, ulcerative colitis, Crohn’s disease

## Abstract

Sarcopenia, defined as a loss of muscle mass and function, is a physiologic factor that has been implicated as a predictor of adverse postoperative outcomes in many older adult populations. However, data related to sarcopenia in older adults with inflammatory bowel disease (IBD) remain limited. Older adults with IBD are particularly vulnerable to adverse postoperative outcomes, in part, due to muscle depletion from systemic inflammation, malnutrition, and reduced physical activity. However, few patients undergo routine muscle evaluation as a part of preoperative assessment. Moreover, cut-off values for measures of sarcopenia in the literature are modeled after non-IBD populations. The lack of standardized measures and values for sarcopenia in the IBD patient population has led to heterogenous findings and a paucity of preoperative risk stratification tools. Therefore, we aim to explore the scope of sarcopenia as a preoperative risk stratification tool among older adults with IBD.

## INTRODUCTION

Inflammatory bowel disease (IBD), which includes both ulcerative colitis (UC) and Crohn’s disease (CD), is a chronic immune-mediated condition of the gastrointestinal tract marked by relapsing inflammation. While IBD is frequently regarded as a disease of the young, older adults are forecasted to represent more than one-third of the IBD patient population in the next decade [1]. This population consists of younger individuals with IBD who are aging, in addition to those with older-onset IBD, with approximately 15% of patients developing IBD after the age of 65 years [2].

Although medical therapy is the foundation of treatment, nearly 20% of patients with UC and 80% with CD will eventually require surgery during the course of disease [3]. This is particularly important in the context of older adults, as these individuals have a higher risk of postoperative complications, as compared to younger adults [4,5]. Notably, a study using the American College of Surgeons National Surgical Quality Improvement Program (ACS-NSQIP) database found that 30-day postoperative mortality was approximately ten-fold higher in older IBD patients as compared to younger IBD patients [4]. Further, in a national study by Fernandez et al., the risk of adverse 30-day postoperative outcomes was 37% in older adults compared to 28% in younger individuals [5]. Although older and younger adults have similar risk factors for adverse postoperative events, data have shown that older adults are more likely to undergo emergency surgery, develop preoperative sepsis, have malnutrition preoperatively, as well as have limited functional status [5]. This discrepancy, in part, stems from initial surgical deferral due to chronological age alone. When surgery is ultimately required, these delays lead to ongoing periods of inflammation, exposure to corticosteroids, malnutrition, and physiologic decline, which all contribute to the higher risk of postoperative adverse events observed among older adults with IBD [4–6]. Improved risk stratification tools, focusing on markers of physiologic aging, have the potential to improve these outcomes [5,6].

Sarcopenia, defined as a loss of muscle mass, strength, and function, is one such physiologic factor that is potentially modifiable and has been linked to adverse postoperative outcomes in older cohorts [7]. In a retrospective study of 170 community-dwelling older adults undergoing emergency surgery, 45% of patients with sarcopenia had postoperative complications as compared to 15% without sarcopenia [8]. However, despite a potential association between ongoing inflammation and the development of sarcopenia, there have been a paucity of data studying this within the older adult IBD patient population. Moreover, among the few studies assessing sarcopenia among all-aged individuals in IBD, there is substantial heterogeneity among measures used to evaluate sarcopenia, as well as cutoffs used to define the presence or absence of sarcopenia [9]. Therefore, in order to lay the foundation for future research, in this review we will: (1) Explore the underlying pathophysiology relating to the development of sarcopenia, focusing on overlapping mechanisms with inflammatory bowel disease, (2) Highlight the association between sarcopenia and adverse operative outcomes in the IBD patient population, (3) Discuss the modifiability of sarcopenia in the preoperative state, and (4) Reflect on avenues for future research.

## DEFINING SARCOPENIA

Sarcopenia, derived from the Greek words “sarx” for flesh and “penia” for poor, was first described as a loss of muscle mass related to aging [10]. The most recent definition of sarcopenia by the European Working Group of Sarcopenia in Older People (EWGSOP), Foundation for the National Institutes of Health (FNIH), and Asian Working Group of Sarcopenia (AWGS) has evolved to include muscle mass and strength, with poor physical performance being an indicator of severity [11–13].

## MEASURES OF SARCOPENIA

While measures of muscle mass and muscle quality can be assessed by an array of modalities, there are limited data regarding which measure is most predictive of adverse postoperative outcomes in the geriatric population. Further, we highlight commonly used measures of muscle strength and function, as well as the limited data available in the older adult IBD patient population.

### Muscle Mass

Muscle mass is an integral component of sarcopenia that can be easily measured through various imaging techniques, such computed tomography (CT), magnetic resonance imaging (MRI), and dual-energy X-ray absorptiometry (DXA). In particular, CT and MRI are regarded as gold standards for non-invasive measures of muscle quality and mass [11]. Muscle mass can be directly measured on CT and MRI by determining the cross-sectional area of either psoas or skeletal muscle at a particular vertebral level, with most studies utilizing L3 [14–16]. Muscle groups found at the L3 level include transversus abdominus, internal oblique, external oblique, psoas, rectus abdominus, quadratus lumborum, and erector spinae muscles [17]. The total area is then divided by the patient’s height (cm^2^/m^2^) to determine a muscle mass index. Skeletal muscle index (SMI) and total psoas index (TPI) are examples of muscle mass indices that have been used in prior studies, though some regard psoas muscle as a minor muscle that has less utility as sole measure of muscle mass [11,18,19]. In particular, SMI was found to be a significant predictor of 2-year and 5-year overall postop survival in patients undergoing head and neck cancer surgery [17]. In another study of 1,513 patients undergoing abdominal surgery for gastrointestinal cancer, the odds of postoperative complications were approximately two times higher among those with sarcopenia, as measured by SMI, as compared to those without [20]. Conversely, in a retrospective study of 85 adults with IBD, sarcopenia as defined by TPI was not associated with adverse postoperative outcomes, further suggesting that psoas muscle may not be representative of overall muscle mass [21].

Both CT and MRI also offer advantages over alternative measures of muscle mass, as they can assess aspects of muscle quality, such as myosteatosis, which is defined as fat infiltration into muscle tissue [22,23]. This offers additional information beyond measures of muscle mass alone and may have the potential to improve our current prognostic tools. More specifically, in a study of 139 patients with esophageal cancer undergoing radical esophagectomy, patients with myosteatosis had a median postoperative survival of 19 months as compared to 57 months among patients without myosteatosis [24]. Additional data in the kidney transplant patient population has found analogous results, with myosteatosis and sarcopenia both significantly increasing the risk of transplant waitlist mortality (62% and 78%, respectively) [25].

In addition to CT and MRI, DXA scans can provide an estimate of appendicular lean mass (ALM), which is obtained from the sum of the upper and lower limb lean mass [22]. Similar to the calculations used for SMI and TPI, ALM can be divided by height to obtain the appendicular lean mass index (ALM/height^2^). Further, lower ALM has also been shown to be associated with adverse postoperative outcomes among the older adult patient population. In a retrospective study of 243 older patients undergoing lumbar spinal surgery, the recovery rate, which compares preop and postop motor and sensory function, was lower in patients with sarcopenia, as measured by DXA, compared to patients without sarcopenia (53.8% vs 68.6%, respectively) [26]. Despite this, DXA scans are often not performed in the preoperative state which can limit their clinical utility in this setting. Further, DXA scans do not provide information about muscle quality, and assessments can be affected by volume status, as water and lean mass tissue can be difficult to differentiate [22,27]. Bioelectrical impedance analysis (BIA) is yet another method recognized by EWGSOP and AWGS for estimating muscle mass [28]. BIA provides an estimate of body composition by calculating the difference between electrical conductivity of different types of tissue, such as bone, muscle, and cartilage [29]. In 153 patients undergoing gastrectomy for gastric cancer, postoperative complications occurred significantly more frequently in patients with sarcopenia as defined by BIA compared to those without (37.5% vs 16.3%) [30].

In the IBD patient population, although limited data exist, studies have primarily employed imaging-based measures (largely CT) to assess muscle mass. In a study of 91 individuals of all ages undergoing IBD-related surgery, the odds of a postoperative infection were five times higher among patients with lower muscle mass as measured by SMI, even after adjusting for age, gender, disease type, and surgical procedure performed [31]. However, these data suffer from inherent limitations, as cutoffs to define the presence or absence of sarcopenia were largely determined from older non-IBD patient populations and applied to an IBD patient population of all ages. In an effort to improve upon current assessments, in a retrospective study of 121 older adults with IBD undergoing disease-related intestinal resection, SMI, measured as a continuous variable, was a significant predictor of adverse 30-day postoperative outcomes [32]. Further, SMI, as measured by CT scan within 90 days of the date of surgery, had a higher area under the curve (AUC) as compared to TPI when assessing risk of an adverse 30-day postoperative outcome; suggesting that SMI may be a better predictor of adverse postoperative outcomes among older adults with IBD as compared to TPI [32].

### Muscle Strength and Physical Performance

In addition to muscle mass, measures of muscle strength and function are integral to assess the presence of sarcopenia. While muscle strength can be evaluated using several different modalities, the most recent AWGS guidelines from 2019 recommend the use of handgrip strength as an indicator of skeletal muscle strength, as it can be measured by a handheld dynamometer in a simple, cost-effective manner [12,33]. Several studies have since used this measure to demonstrate its ability to predict adverse postoperative outcomes among older adults undergoing surgery. In particular, in a retrospective study of 327 individuals (mean age: 68.16 ± 10.25) with gastric cancer undergoing gastrectomy, 17.5% of individuals with low handgrip strength had a postoperative infection as compared to 1.8% among those with higher handgrip strength [34]. Handgrip strength has also been reported as a tool to predict postoperative functional status in older adults. For instance, in a prospective study of 63 older adults undergoing surgery after hip fracture, handgrip strength was a significant predictor of early postoperative ambulation [35]. Further, older adults who began ambulating within three days postoperatively had a significantly lower rate of postoperative complications (0%) as compared to those who required more than three days to begin ambulating (32%) [35]. This emphasizes the importance that grip strength assessments can have in the preoperative state; both as a tool for prognostication, as well as a tool to focus resources and future prehabilitation (strength training) efforts.

Physical performance can also be assessed in a variety of ways, including the 6-minute walk, timed-up-and-go test, and Short Physical Performance Battery (SPPB), which provides measures of function, mobility, and balance. In the non-IBD patient population, a study of 364 older adults undergoing lung resection surgery found that the odds of postoperative pulmonary complications were approximately nine times higher in patients with low SPPB scores as compared to those with higher scores [36]. While prospective measures of physical strength and function have been evaluated in the older adult IBD population, these measures are yet to be studied in the preoperative setting [37]. Additionally, the Strength, Assistance with walking, Rising from a chair, Climbing stairs, and Falls (SARC-F) questionnaire is another measure of physical performance that is often used in clinical practice to screen patients at risk for sarcopenia [38,39]. In a study of 129 adults with IBD, SARC-F scores were significantly lower in patients with IBD than in age- and sex-matched healthy controls [40].

## PATHOGENESIS OF SARCOPENIA IN IBD

**Figure 1.**
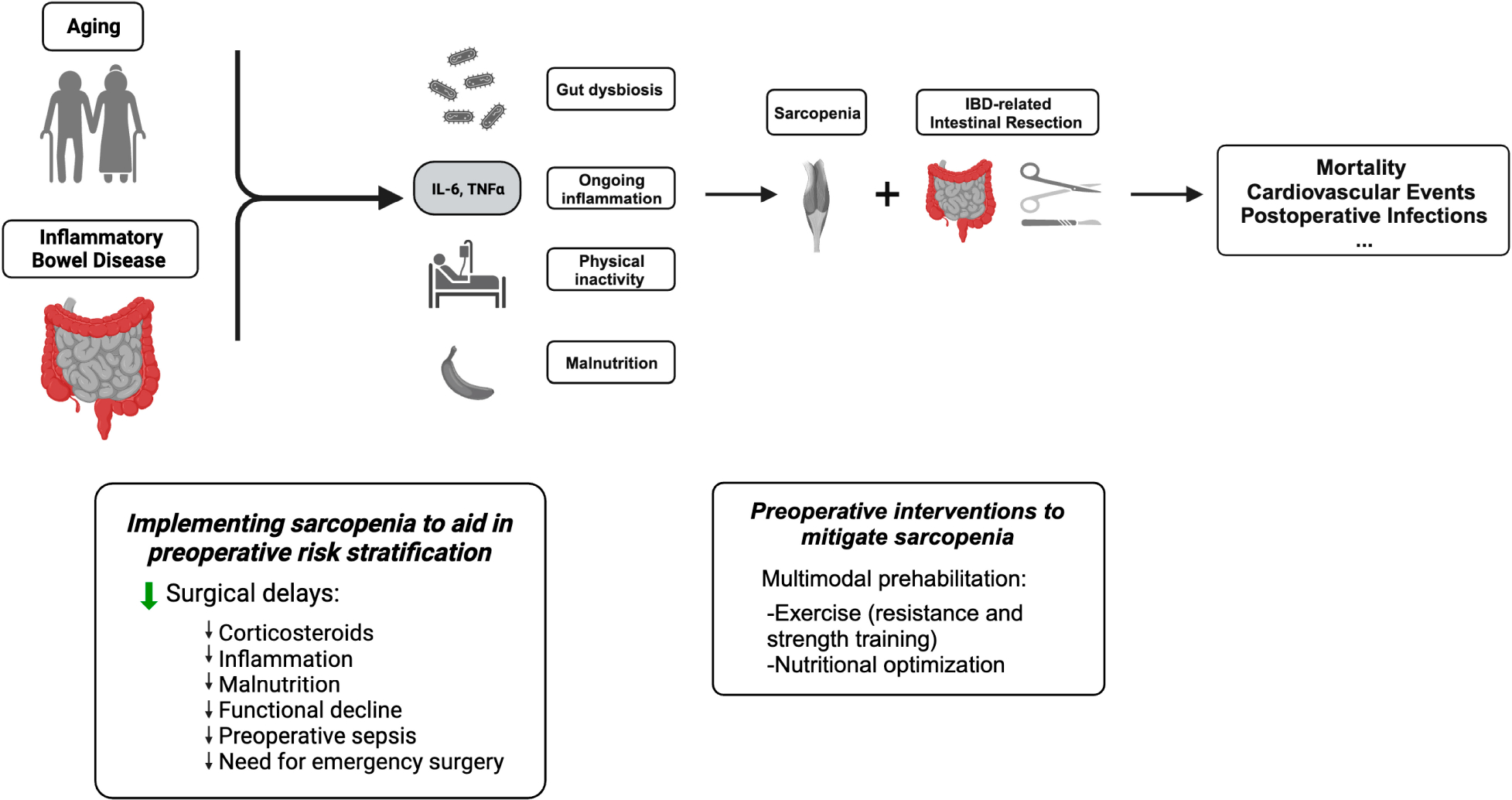
A visual model depicting the relationship between inflammatory bowel disease (IBD), aging, and sarcopenia as well as how identification and treatment of sarcopenia may mitigate adverse postoperative outcomes among the older adult IBD patient population. Figure was created with BioRender.com.

The pathogenesis of sarcopenia involves a complex interplay of processes, and has significant overlap with the underlying pathophysiology of IBD. More specifically, chronic inflammation, gut dysbiosis, malnutrition, and reduced physical activity, which are common among individuals with IBD, can all contribute to the development of sarcopenia (Figure 1) [41].

Inflammation in particular has been linked to the development of sarcopenia, with data showing that individuals with sarcopenia have higher levels of circulating TNF-α and interleukin (IL)-6 [42–44]. Although aging itself is a chronic inflammatory process that can lead to protein breakdown and reduced muscle protein synthesis, a similar cascade of events is seen in IBD, suggesting that the presence of IBD may potentiate the development of sarcopenia. More specifically, among individuals with IBD, there is an upregulation TNF-α, IL-6, and interferon (IFN)γ, which all contribute to a higher overall systemic inflammatory burden and can lead to the development of sarcopenia [43]. Notably, increased TNF-α levels have been associated with muscle impairment [41]. We see evidence of this, as a cross-sectional study demonstrated that the prevalence of sarcopenia among patients with IBD was significantly higher (28%) as compared to that seen among healthy controls (1%) [45]. Further, in a study of 90 individuals with IBD, those with sarcopenia had higher levels of C-reactive protein (CRP), a circulating inflammatory marker, as compared to those without sarcopenia [46]. It is important to note, however, that disease severity which was not controlled for can also contribute to the increased CRP levels observed [47].

In addition to ongoing inflammation contributing to the development of sarcopenia, the gut microbiome has also been implicated in maintaining skeletal muscle homeostasis [41]. More specifically, the gut microbiota regulates various physiologic processes, including nutrient absorption and inflammation [48]. Age-related changes in the gut microbiome, such as reduced microbial diversity, contributes to dysfunction of the gut mucosal barrier, leading to increased intestinal permeability and translocation of microbial byproducts [48]. As a result, circulating endotoxins induce systemic inflammation, and, eventually, increase skeletal muscle catabolism [49]. Separate from aging, this process also occurs among individuals with IBD, as gut dysbiosis is a hallmark of IBD [41]. Thus, with an increase in bacterial species that disrupt the intestinal barrier, there is a shift towards a pro-inflammatory state, which leads to muscle breakdown and impairment [50,51]. We see evidence of this in a cross-sectional study of 88 patients with CD, where the mean handgrip strength was reduced for patients with CD (33.1 kg) as compared to that of age and BMI-matched healthy controls (37.14 kg) [52].

Malnutrition is another factor related to the development of sarcopenia that is highly prevalent among individuals with IBD. In particular, an observational study using the Nationwide Readmissions Database found that hospitalized patients with IBD were approximately three times more likely to have protein-calorie malnutrition as compared to hospitalized patients without IBD [53]. In patients with IBD, malnutrition manifests due to a host of factors, including reduced food intake, malabsorption, intestinal bacterial overgrowth, chronic diarrhea, and medications such as glucocorticoids [54]. Further, active intestinal inflammation in IBD alters intestinal permeability and reduces the contact time between nutrients and the intestinal surface, leading to malabsorption and decreased amino acid absorption [54,55]. Diminished amino acid uptake leads to decreased muscle protein synthesis, and thus, the development of sarcopenia [56]. Additionally, patients with IBD are frequently deficient in Vitamin D, a fat-soluble vitamin that plays an important role in preventing mitochondrial dysfunction and maintaining skeletal muscle function [57,58].

Physical inactivity has also been implicated in the pathogenesis of sarcopenia, with low energy expenditure contributing to low-grade systemic inflammation that exerts a catabolic effect on muscle mass [59]. Among patients with IBD, active disease, including abdominal discomfort and frequent need to evacuate, can limit ability to ambulate far distances, further contributing to the development of sarcopenia. Additionally, among individuals with IBD, fatigue is highly prevalent (~70%), which further contributes to physical inactivity, and the decline of overall muscle mass, strength, and function [60].

## SARCOPENIA AS A PREOPERATIVE RISK STRATIFICATION TOOL

Sarcopenia has been shown to be a significant predictor of adverse postoperative events among many older adult populations. Among older adults undergoing surgery for hip fracture repair, one-year postoperative mortality was three times higher in those with evidence of sarcopenia as compared to those without [61]. Similar results are seen among older adults undergoing gastrointestinal surgery, as lower muscle mass conferred a reduced 1-year overall postop survival of 63.6% as compared to 84.3% among older adults with a higher SMI [62]. When pooling available data in a systematic review of older adults undergoing gastrointestinal resection, the odds of a major postoperative complication were noted to be four times higher among patients with sarcopenia as compared to those without [63].

Data investigating the association between sarcopenia and postoperative outcomes in IBD however, remain limited. In a retrospective review of 178 patients of all ages with IBD, the presence of sarcopenia was associated with a higher risk of requiring blood transfusions postoperatively, as well as ICU admission and postoperative sepsis [64]. Similarly, in a study of 114 CD patients undergoing bowel resection, patients with sarcopenia as measured by SMI had a higher risk of a major postoperative complication as compared to those without sarcopenia (15.7% vs 2.3%, *p* = 0.03) [65]. Although there has been heterogeneity among measures of muscle mass utilized in patients with IBD, a systematic review found that radiological assessment of skeletal muscle mass correlated with an increased rate of major postoperative outcomes among individuals with IBD [66]. This suggests that sarcopenia may be used to help risk stratify older adults in the preoperative state, identifying those who are both at higher and lower risk of an adverse postoperative outcome. Further, specifically among older adults with IBD, a retrospective study of 121 older adults with IBD undergoing disease-related intestinal resection found that the odds of a major postoperative event decreased by 13% for each one unit increase in SMI, again suggesting the importance of measures of muscle in the preoperative setting [32].

Sarcopenia, as defined by muscle mass, has also been explored as an independent predictor for the need for intestinal surgery among individuals with IBD. For example, among 254 adults with acute severe UC, the presence of sarcopenia as determined by SMI, was associated with an increased need for colectomy within one year of initial hospitalization for acute severe UC (22% required colectomy among those who were sarcopenic vs 7% among those who were not sarcopenic) [67]. In a similar study of 72 patients with IBD, those with sarcopenia, as measured by SMI, had a lower cumulative surgery-free rate as compared to those who were not sarcopenic [68].

Although these findings emphasize the importance of assessing sarcopenia preoperatively in IBD, there are also significant limitations to our current data. This, in part, is due to the reliance on retrospective measures of muscle mass without the inclusion of measures of muscle strength and function. Further, as previously noted, cut-off values used to determine the presence/absence of sarcopenia are largely based upon reference values from older adults without IBD and applied to individuals with IBD of all ages, making overall data interpretation challenging. Future work building upon this foundation is therefore necessary to advance our understanding of the association between sarcopenia and postoperative outcomes among all adults with IBD, with a particular focus on older adults.

Additionally, frailty, which is defined as a decrease in physiologic reserve, has also been associated with the development of adverse postoperative outcomes among individuals with IBD [69]. While there is considerable overlap between sarcopenia and frailty, they remain distinct entities, and the operative risk associated with each remains to be explored among the older adult IBD patient population [70]. Further, as muscle strength and function are part of the frail phenotype, it is possible that much of the increased postoperative risk observed among frail individuals is driven by the presence of sarcopenia [69]. We see evidence of this in a recent nationwide study, in which the greatest risk for an adverse postoperative outcome among older adults with IBD was driven by a limitation in functional status [5]. Future work exploring this, however, needs to be undertaken.

## SARCOPENIA AS A MODIFIABLE RISK FACTOR

Sarcopenia can not only aid in risk stratification, facilitating preoperative clinical decision making, but also is of particular interest because of its potential to be modified. Prehabilitation is a preoperative intervention that involves exercise training, nutritional optimization, and psychological support [71]. This multifaceted model has the potential to improve functional status in older adults undergoing surgery. In a systematic review and meta-analysis of patients undergoing colorectal surgery, nutritional prehabilitation alone or in combination with an exercise program reduced the length of hospital stay by two days [72]. In another study of adults undergoing colorectal cancer surgery, the number of severe postoperative complications in patients who participated in a 4-week prehabilitation program was 17.1% versus 29.7% among patients who were treated with standard of care preoperatively [73].

Preoperative interventions have also been investigated in IBD cohorts. In a randomized cross-over trial of 17 patients with quiescent UC and CD, patients who underwent a combined aerobic and resistance training program experienced a median *increase* of 1.59 kg in total lean tissue mass as compared to a *decrease* of 1.38 kg in the control group [74]. In a similar study, 45 patients with quiescent or mildly active CD were randomly distributed to a control, endurance, or muscle training group, with patients in both exercise groups experiencing an increase in handgrip strength [75]. Further, among individuals with IBD, moderate-intensity exercise has been shown to decrease pro-inflammatory cytokines, leading to improvements in muscle composition [76]. In another study of IBD patients undergoing elective disease-related surgery, nutritional prehabilitation was associated with a significant improvement in weight, body mass index, and lean mass during the preoperative phase [77]. These studies highlight the potentially modifiable nature of sarcopenia in IBD, particularly in the preoperative state. However, the clinical impact of this improvement in muscle mass, strength, and/or function remains unknown.

Additionally, limiting IBD-related inflammation through adequate disease control also has the potential to improve sarcopenia. For instance, infliximab therapy (IFX), an anti-TNF agent used to control disease-related inflammation, has been found to prevent the activation of biochemical pathways that potentiate muscle breakdown by proteolysis [78]. In a prospective study of 19 CD patients with an acute disease flare, IFX therapy was associated with a significant and progressive increase in muscle volume and strength [78]. Further, in a cross-sectional analysis of older adults with IBD, disease activity itself was associated with a significant decrease in physical capacity [37]. This is likely driven by an upregulation of IBD-related proinflammatory factors (e.g., TNFα, IL-6), which have also been shown to impact muscle protein metabolism [43]. Beyond medical management, surgery to control disease-related inflammation has also been shown to improve measures of muscle mass. More specifically, in a retrospective study of 14 UC patients undergoing colectomy, there was a significant increase in SMI post colectomy (49.92 cm^2^/m^2^ pre-surgery vs. 61.52 cm^2^/m^2^ post-surgery) [79].

## FUTURE DIRECTIONS

As the number of older adults with IBD is rapidly increasing, a growing number of older adults will require IBD-related intestinal resection in the next decade. However, with a lack of adequate preoperative risk stratification tools, older adults often have surgery deferred based upon chronological age alone. However, when surgery is required, these delays increase the risk of malnutrition, physical decline, the need for corticosteroids, the development of preoperative sepsis, and the ultimate need for emergency surgery; contributing to the higher risk of adverse postoperative outcomes observed in older adults with IBD.

Assessing sarcopenia preoperatively, however, has the potential to change this paradigm, as it appears to play a critical role in determining postoperative clinical outcomes. This is true particularly in IBD, where an amalgamation of chronic inflammation, malnutrition, and reduced physical activity all contribute to a potentially greater depletion of muscle mass, strength, and function. However, while evidence from prior studies suggests that sarcopenia is a significant predictor of adverse postoperative outcomes among all adults with IBD, variability in cut-off values, a reliance on varying retrospective measures of muscle mass, an exclusion of muscle strength and function, and a paucity of data among older adults with IBD all pose a considerable challenge for preoperative risk stratification among the older adult IBD patient population. Future prospective studies are therefore needed, focusing on the older IBD patient population undergoing intestinal resection in order to determine (1) the measures of muscle mass, strength and function that are most predictive of adverse postoperative outcomes, (2) the postoperative risk attributable to the presence of sarcopenia, and (3) the ability for sarcopenia to be modified in the preoperative state. This has the potential to improve preoperative risk stratification, thereby reducing surgical delays among older adults with IBD who are at lower preoperative risk, as well as both identifying older adults who are at higher risk for an adverse postoperative outcome and focusing future prehabilitation efforts (Table 1).

## CONCLUSIONS

Sarcopenia is a significant predictor of adverse postoperative outcomes among older adults, however data within the older adult IBD patient population are lacking. Further understanding the risk that sarcopenia confers among older adults undergoing IBD-related intestinal resection is essential, both to reduce surgical delays among those at lower risk and to target preoperative interventions among those at highest risk for an adverse postoperative outcome. This has the potential to improve clinical outcomes for the growing population of older adults with IBD.

## Figures and Tables

**Table 1.** Knowledge gaps and areas of focus for future research pertaining to sarcopenia among older adults with inflammatory bowel disease (IBD).

Knowledge Gaps	Areas of Focus for Future Research
Lack of standardized measures and values for sarcopenia among older adults with IBD	• Prospectively determine and validate measures of muscle mass, strength, and function that are most associated with adverse clinical outcomes among older adults with IBD • Determine IBD-specific reference and cutoff values for various measures of muscle mass, strength, and function among older adults with IBD
Limited data on optimal interventions for sarcopenia among older adults with IBD	• Perform studies to identify effective interventions to prevent and manage sarcopenia and to further understand the role of sarcopenia as a modifiable risk factor among older adults with IBD • Generate tailored treatment strategies that consider factors such as disease subtype (CD vs. UC), comorbidities, and age (older vs. younger adults with IBD) • Assess the impact of sarcopenia on the quality of life in patients with IBD, including functional status
Understand the association between sarcopenia and postoperative outcomes in older adults with IBD	• Investigate the role of sarcopenia as a predictor of adverse clinical outcomes, postoperative outcomes, and the need for surgical intervention in older adults with IBD
Measures of sarcopenia are not routinely employed to preoperatively risk stratify patients with IBD	• Raise clinician awareness about the association between sarcopenia and adverse postoperative outcomes in patients with IBD to promote early interventions and improve postoperative outcomes • Incorporate simple, cost-effective measures of sarcopenia, such as handgrip strength, into routine preoperative evaluation of patients with IBD

## Data Availability

No data were generated from the study.

## ABBREVIATIONS

ACS-NSQIP: American College of Surgeons National Surgical Quality Improvement Program
ALM: appendicular lean mass
AWGS: Asian Working Group of Sarcopenia
BIA: bioelectrical impedance analysis
CRP: c-reactive protein
CD: Crohn’s disease
CT: computed tomography
DXA: dual-energy X-ray absorptiometry
EWGSOP: European Working Group of Sarcopenia in Older People
FNIH: Foundation for the National Institutes of Health
IBD: inflammatory bowel disease
IFN: interferon
IFX: infliximab
IL: interleukin
MRI: magnetic resonance imaging
SARC-F: Strength, Assistance with walking, Rising from a chair, Climbing stairs, and Falls
SMI: Skeletal Muscle Index
SPPB: Short Physical Performance Battery
TPI: Total Psoas Index
TNF: tumor necrosis factor
UC: ulcerative colitis
